# Microbial derived antimicrobial peptides as potential therapeutics in atopic dermatitis

**DOI:** 10.3389/fimmu.2023.1125635

**Published:** 2023-01-25

**Authors:** Aaroh Anand Joshi, Marc Vocanson, Jean-Francois Nicolas, Peter Wolf, Vijaykumar Patra

**Affiliations:** ^1^ Department of Dermatology and Venereology, Medical University of Graz, Graz, Austria; ^2^ Centre International de Recherche en Infectiologie, Institut National de la Santé et de la Recherche Médicale, U1111, Université Claude Bernard Lyon 1, Centre National de la Recherche Scientifique, UMR 5308, Ecole Normale Supérieure de Lyon, Université de Lyon, Lyon, France; ^3^ Department of Allergology & Clinical Immunology, Lyon-Sud University Hospital, Lyon, France; ^4^ BioTechMed Graz, Graz, Austria

**Keywords:** antimicrobial peptides, atopic dermatitis, autoinducing peptides, bacteriocins, skin microbiome, *Staphylococcus aureus*

## Abstract

Atopic dermatitis (AD) is a common chronic inflammatory skin disease that significantly affects the patient’s quality of life. A disrupted skin barrier, type 2 cytokine-dominated inflammation, and microbial dysbiosis with increased *Staphylococcus aureus* colonization are critical components of AD pathogenesis. Patients with AD exhibit decreased expression of antimicrobial peptides (AMPs) which is linked to increased colonization by *Staphylococcus aureus*. The skin microbiome itself is a source of several AMPs. These host- and microbiome-derived AMPs define the microbial landscape of the skin based on their differential antimicrobial activity against a range of skin microbes or their quorum sensing inhibitory properties. These are particularly important in preventing and limiting dysbiotic colonization with *Staphylococcus aureus*. In addition, AMPs are critical for immune homeostasis. In this article, we share our perspectives about the implications of microbial derived AMPs in AD patients and their potential effects on overlapping factors involved in AD. We argue and discuss the potential of bacterial AMPs as therapeutics in AD.

## 1 Atopic dermatitis and its features

Atopic dermatitis (AD), also called atopic eczema, is one of the most common skin diseases in humans (1). The prevalence of AD ranges from 15% to 25% in children and from 7% to 10% in adults (2). AD shows remarkable heterogeneity in clinical presentation, but in most patients, the disease manifests as a condition with extensive eczematous lesions with red, dry, scaly patches that are intensely itchy. Patients with AD often suffer from sleep deprivation and depression, which significantly affects their quality of life (3, 4). The prevalence of AD varies by geographic location and has nearly doubled to tripled in developed countries in recent decades. One explanation for it is provided by the “biodiversity hypothesis”, i.e., that a reduced contact to natural environments in early life does lead to a failure of enrichment in the human microbiome, disturbs immune balance and leads in turn to allergy and inflammatory disorders (5–7).

Although the pathogenesis of AD is complex and not fully understood, three key features are involved in its development. These include (i) disruption of epidermal barrier function, (ii) an excessive immune response mediated by type 2 cells, including Th2, and innate lymphoid cells (ILCs) cells, and (iii) skin microbial dysbiosis with excessive growth of *Staphylococcus aureus* (*S. aureus*). AD patients exhibit altered stratum corneum lipids composition, decreased moisture content, and increased permeability to environmental molecules in both lesional and nonlesional skin (8, 9). Defects in structural barrier proteins (e.g., filaggrin) and the itch-scratch cycle are among the major causes of the observed barrier deficiencies in AD (10, 11). Epidermal barrier disruption leads to excessive secretion of damage-associated molecular patterns (DAMPs) and alarmins such as thymic stromal lymphopoietin (TSLP), IL-1ß, IL-25, and IL-33. Alarmins promote the secretion of cytokines IL-13 and IL-5 by immune cells such as dendritic cells and ILCs, which further promote a type 2 cell-mediated immune response (12). Increased allergen permeation, along with a Th2-cell mediated immune response lead IgE isotype switching (13); a hallmark of chronic recurring AD. Skin microbiome, the third feature involved in the pathogenesis of AD will be discussed in detail in the next sections.

## 2 Skin microbiome in AD

The skin harbours millions of bacteria, fungi, viruses, archaea, and skin mites that together form the skin microbiome. Bacteria constitute the most abundant kingdom in the skin microbiome with the major genera being *Cutibacterium*, *Corynebacterium*, and *Staphylococcus* (14, 15). The composition and abundance of these genera depend upon the skin site, physiology, and microenvironments (sebaceous, dry, and moist). Skin microbiome is now known to be an imperative component of skin homeostasis maintenance, which includes development of skin’s barrier functions (16), immune system, breakdown of natural products and protection against invading pathogens (14, 17).

### 2.1 Role of *S. aureus* in AD

The implications of microbial shifts correlate with several dermatological diseases most notably, AD (18). The prevalence of *S. aureus* in patients with AD is approximately 20 times higher than the skin of healthy controls. The lesional skin of AD patients shows higher prevalence of *S.aureus* (up to 70%) than nonlesional skin of the same patients (39%) (19). A positive correlation was found between *S. aureus* density on lesional and nonlesional skin and disease severity (20). Moreover, the higher abundance of *S. aureus* in AD patients is independent of age group, ethnicity, and geographic location (21, 22).

**Figure 1:**
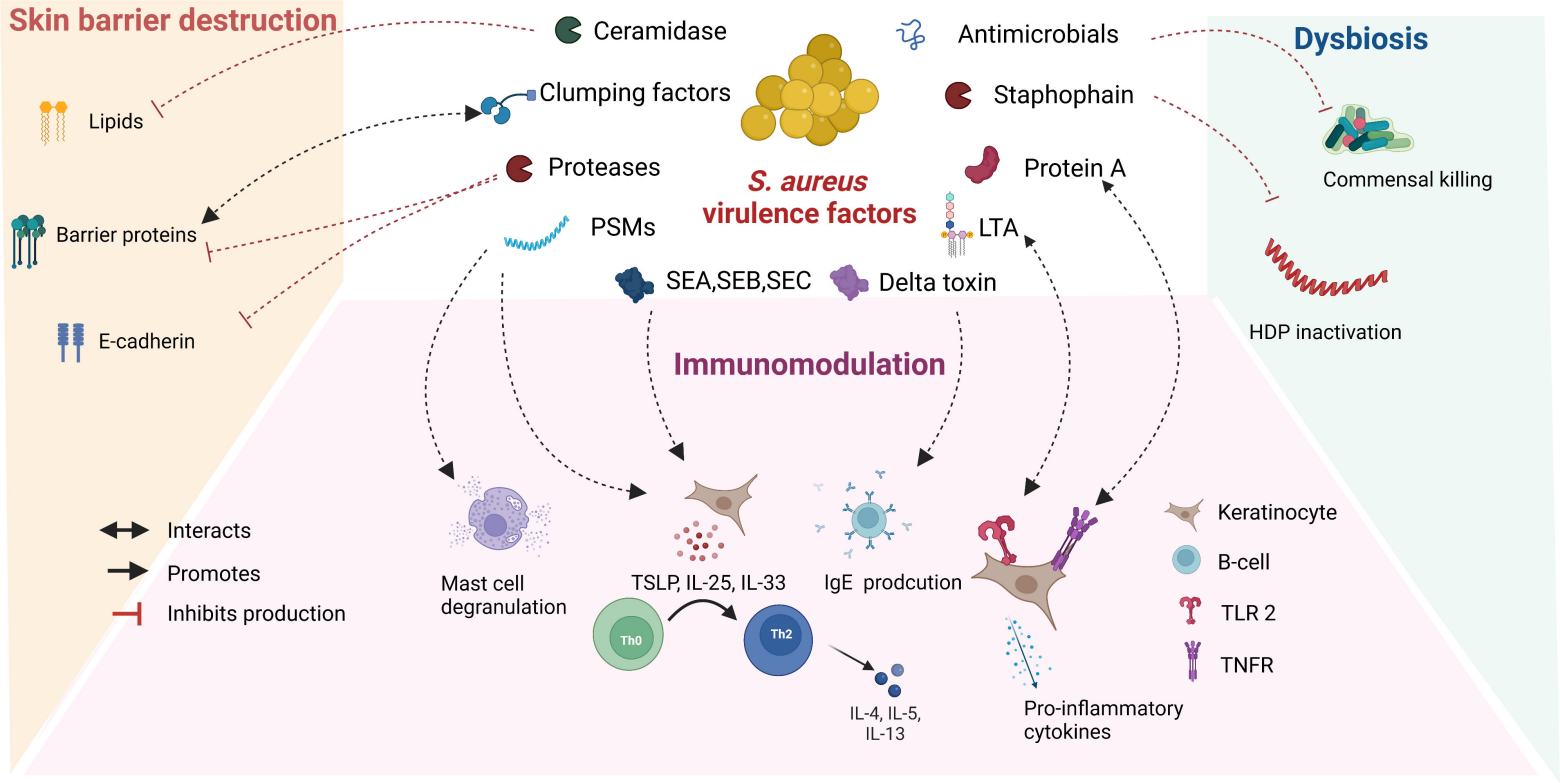
Role of *S. aureus* secreted virulence factors in the pathogenesis of atopic dermatitis: *S. aureus* secretes several virulence factors, which interact with overlapping features involved in the pathogenesis of AD. Abbreviations: VF: virulence factors, PSMs: Phenol soluble modulins, SE: staphylococcal enterotoxins, LTA: Lipoteichoic acid, HDP: Host defence peptide, TSLP: Thymic stromal lymphopoietin, TLR: Toll like receptor, TNFR: Tumor necrosis factor receptor.

*S. aureus* secretes several metabolites, also known as virulence factors, which are responsible in part for the proinflammatory activity and barrier destruction in AD lesions (**Figure 1**). The enzyme ceramidase secreted by *S. aureus* lowers lipid and fatty acid levels and makes the skin permeable to allergens (23). Lower fatty acid levels also lead to decreased formation of phospholipid hydrolysis products in sebum and sweat, which increase skin surface pH and further promote *S. aureus* growth. Cell surface proteins of *S. aureus* such as clumping factors A and B and fibronectin-binding proteins support attachment to the uppermost skin, the stratum corneum (24, 25). *S. aureus* is known to secrete several proteases and is also able to induce protease secretion from host (26). Staphophain, inactivates host defence peptides, thus promoting *S. aureus* colonization (27). Proteases also promote the permeation of allergens through the stratum corneum by acting on barrier proteins and tight junctions (28). Alpha toxin secreted by *S. aureus* is cytotoxic to keratinocytes and alters the integrity of E-cadherin, compromising barrier function (29). Staphylococcal enterotoxins (SE) (SEA, SEB, and SEC), phenol-soluble modulins, and lipoproteins target key immune pathways and create a pro-inflammatory environment characteristic of patients with AD (29). The mechanisms of virulence factors secreted by *S. aureus* that play a role in the pathogenesis of AD have been described in detail elsewhere (30–32).

So far, there appears to be no evidence linking the presence of a single virulence factor to AD severity*. S. aureus* strains isolated from AD skin differ from those isolated from healthy individuals (33). Fleury et al. showed that the frequency of isolation of *S. aureus* strains belonging to clonal complex (CC) 1 is higher than that of strains isolated from the nasal cavity of healthy children, which have a higher frequency of isolation of CC30 (24). Byrd et al. performed shotgun metagenome sequence analysis of the skin microbiome at the strain level in patients with AD throughout the disease course. They found that the severity of AD is strain-specific, with isolated strains from severe AD lesions being phylogenetically similar. This suggests that specific combinations of virulence factors expressed by certain strains may be responsible for the pathogenicity of *S. aureus* observed in AD (34).

*S. aureus* modulates the production of virulence factors necessary for its survival by sensing environmental factors such as cell density. This phenomenon is known as quorum sensing. The accessory gene regulator (agr) quorum sensing system is one of the best studied quorum sensing systems in *S. aureus*. The agr quorum sensing system recognizes cognate autoinducing peptides which are short peptides of 7-12 amino acids containing a cyclic thiolactone at the C- terminus (35). Interestingly, several virulence factors such as superantigens, lipases, and proteases involved in the pathogenesis of AD are also controlled by the agr quorum-sensing system (35, 36). Nakamura et al. demonstrated that agr virulence is essential for the epithelial degradation observed in AD. Moreover, the probability of developing AD is higher when *S. aureus* with a functional agr virulence system is present in childhood (37). These studies suggest that inhibition of agr quorum-sensing in addition to inhibition of *S. aureus* growth may be a promising therapeutic target in AD.

### 2.2 Role of commensal microbiome in AD

AD skin has lower microbial diversity compared to healthy individuals. This is associated with a lower abundance of the genera *Streptococcus*, *Corynebacterium*, and *Cutibacterium*, as well as members of the commensal *Staphylococci* with anti-*S. aureus* activity (38, 39). These bacterial communities play a critical role in the immune response to pathogens. Ridaura et al. showed that the genus *Corynebacterium* can promote IL-23 signalling and induce IL-17A γδT cells in the dermis, which recruit immune cells (40). At the species level, *Cutibacterium acnes* associated with healthy skin were shown to activate the release of extracellular traps from specialized Th17 subsets (41). Coagulase negative *Staphylococci* (CoNS) such as *Staphylococcus cohnii* activate the host steroid pathway and promote immunosuppression (42), while application of *Staphylococcus epidermidis* to mouse skin showed enhanced innate protection against *Candida albicans* by upregulating Th17 immune mediators such as S100A8 and S100A9 (43). Commensal microbes also play a critical role in epidermal barrier development and surface pH regulation (16, 44). For example, *Roseomonas mucosa* secretes glycerophospholipids which induce host epithelial repair by enhancing the cholinergic activation *via* TNFR2 signalling and *Staphylococcus epidermidis* increases the production of skin ceramides (45, 46), while *Cutibacterium acnes* secretes a lipase that converts triacylglycerols contained in sebum to propionic acid, which contributes to the acidification of the skin surface; a factor that limits the growth of *S. aureus* (47). Notably, commensals directly provide colonization resistance to pathogenic bacteria including *S. aureus* by secreting certain metabolites that inhibit their growth or virulence factor production. The most promising of these metabolites are bacterial-derived AMPs and are discussed in detail in later sections. Additionally, the host-derived AMPs also play a significant role in defining the skin microbiome composition and are discussed briefly as well.

## 3 Host-derived AMPs/host defence peptides in AD

Host-derived AMPs, also known as host defence peptides (HDPs), are mostly cationic peptides with a molecular weight of less than 10 kDa (48–51) but also include a few classes with larger molecules than 10 kDa (52, 53). These peptides are present on epithelial surfaces such as the oral mucosa, vaginal epithelia, skin etc. HDPs are either constitutively produced by keratinocytes and immune cells or induced in response to stimuli such as pathogen-associated molecular patterns (PAMPs) or inflammatory cytokines. Some important classes of HDPs include RNases, defensins, cathelicidins, dermcidin, and S100 class peptides (51, 54, 55).

HDPs show broad-spectrum but variable antimicrobial activity against different pathogens and members of the commensal microbiome. For example, RNAse7 showed higher inhibitory activity against *E. Coli* and *Cutibacterium acnes* compared to *S. aureus* (56). Interestingly, in the context of the vaginal microbiome, certain commensal bacteria have been shown to use constitutively expressed peptides (S100A7 and Elafin) as amino acid sources to ensure their survival (57). This phenomenon is most likely also found in skin epithelia but has not yet been studied. HDPs are well-known for their immunomodulatory and barrier-improving properties and play a critical role in the pathogenesis of diseases such as psoriasis and polymorphic light eruption underscoring the versatile role of HDPs in skin physiology (58–60).

Atopic skin exhibits lower levels of certain HDPs such as dermcidin, human beta-defensin-2 (hBD-2), human beta-defensin-3 (hBD-3), and cathelicidin, as well as increased expression of RNase7 and S100A7 (61–64). Moreover, a higher abundance of *S. aureus* in atopic skin and during disease flare-ups has also been linked to defective HDP expression (18, 65). Th2 cytokine activity and lower vitamin D levels are thought to be responsible for the altered HDP expression observed in AD (66–68). Interestingly, AD treatments such as UVB phototherapy improve expression of certain HDPs which has led researchers to question whether HDPs could potentially be used as a therapy in AD (69–71). This potential was recently indicated by Peng et.al, who showed that subcutaneous injection of hBD-3 in mice with AD alleviated inflammation through its barrier-improving properties (72). Several detailed reviews on host defence peptides are published during the last five years (58, 71, 73, 74).

## 4 Bacterial AMPs in AD

**Figure 2:**
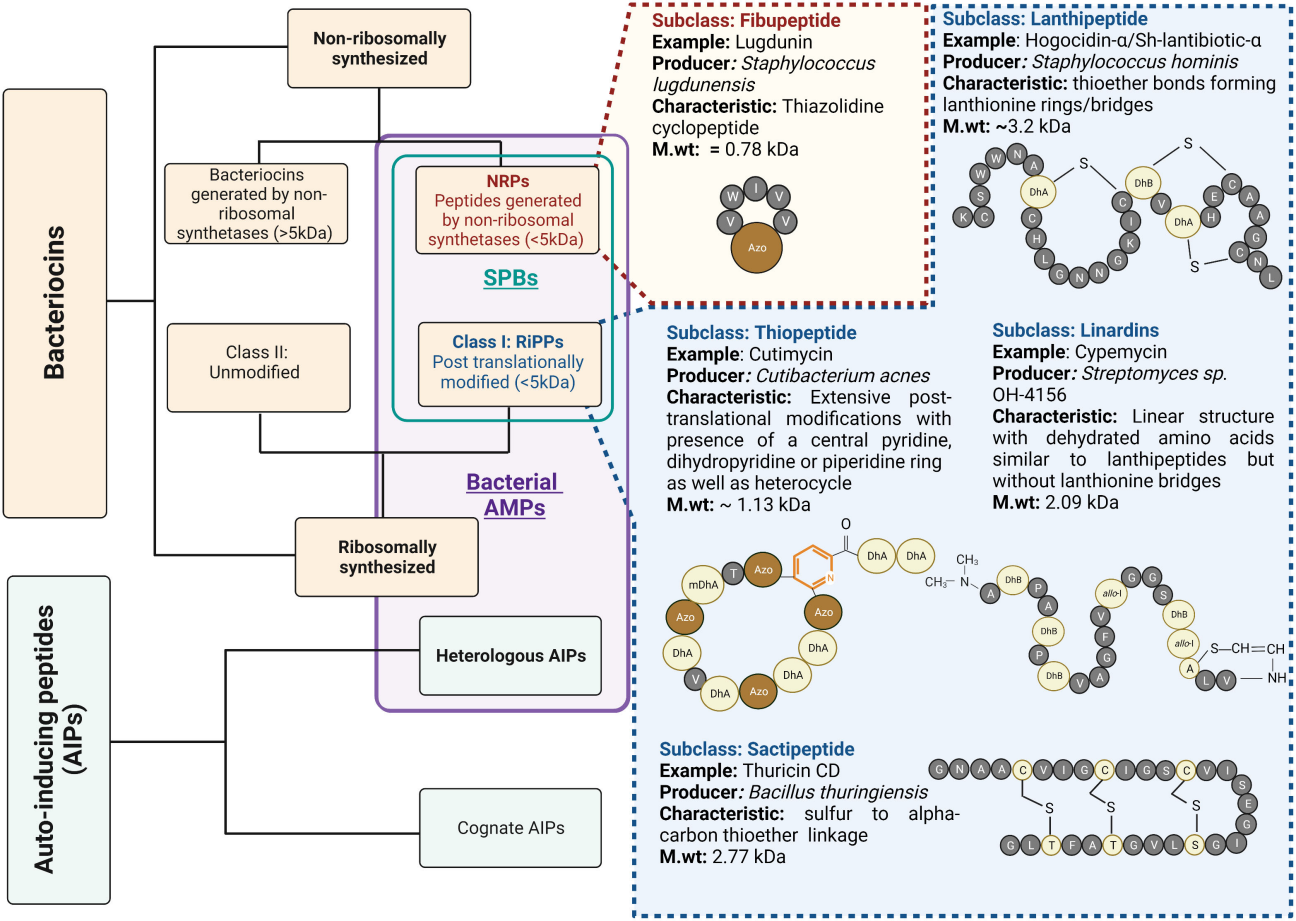
Bacterial AMPs and structural variety of short peptide bacteriocins: Bacterial AMPs include Short Peptide Bacteriocins (SPBs) with molecular weight less than 5kDa and Quorum quenching peptides, which are heterologous autoinducing peptides. The subcategory SPBs also includes the class I bacteriocins which undergo post-translational modifications of amino acids, also known as RiPPs, and the peptides synthesized by non-ribosomal synthetases (NRPs) with molecular weight less than 5kDa. SPBs constitute several subclasses based on their structural characteristics. The figure enlists some important classes with examples. Standard amino acids are described as single letter code with grey colour balls. The colour light yellow represents post-translationally modified amino acids. The prefix “Dh” stands for Dehydro and prefix “allo” represents stereo isomer of amino acid. The pyridine ring is coloured in orange. The azole moieties are indicated by abbreviation “Azo” and brown colour balls.

Bacterial AMPs are small peptide substances usually restricted to short peptide bacteriocins that inhibit microorganisms by physically disrupting the cell membrane or interact with the intracellular components crucial for bacterial survival (75). Recently, bacteriocins and peptide molecules that inhibit the quorum-sensing activity of competing bacteria have gained increasing attention. The inhibitory effect on the quorum-sensing activity of effector bacteria results in reduced virulence, giving the producer a competitive advantage. These peptides are known as heterologous autoinducing peptides (AIPs) and are considered a class of bacterial AMPs in this review (**Figure 2**).

### 4.1 Short peptide bacteriocins and their clinical potential

Bacteriocins are structurally diverse, and there are several classification systems that make them difficult to follow. Many authors restrict the term bacteriocin to ribosomally synthesized peptide antimicrobials with a molecular weight of less than 10 kDa (76, 77). They further divide bacteriocins into class I bacteriocins, which are post-translationally modified, and class II bacteriocins, which are unmodified. These class I bacteriocins are also referred to as ribosomally synthesized and post translationally modified peptides (RiPPs). Recently, however, the term bacteriocin has also been used to refer those AMPs generated by non-ribosomal synthetases (78–80). In the following, we will only discuss about the peptides with a molecular weight of less than 5 kDa, belonging to the group of RiPPs, and those peptides with a molecular weight of less than 5 kDa generated by non-ribosomal synthetases (NRPs). They are collectively known/described as short peptide bacteriocins (SPBs) (**Figure 2**).

Members of SPBs possess potent narrow spectrum antibacterial activity at nanomolar concentrations, and have lower chances of resistance development (81). The important classes belonging to SPB group, and their structural details are shown in **Figure 2**. SPBs inhibit bacterial growth by several mechanisms. For example, the lantibitoic nisin binds to the lipid II, which is responsible for peptidoglycan synthesis, thus interfering with cell wall biosynthesis of target bacteria. In addition, its C-terminal region leads to the formation of pores that result in membrane disruption and efflux of bacterial metabolites necessary for growth (82, 83). Bottoromycins and Thiopeptides such as thiostrepton and micrococcin inhibit bacterial protein synthesis by binding to the 50s ribosomal subunit. Glycocins (e.g., sublancin 168) bind to and inhibit the function of the glucose phosphotransferase system and mechanosensitive channel (MscL) while Sactipeptides e.g., Ruminococcin C inhibits RNA polymerase (84).

The antimicrobial activity of SPBs is mainly directed against closely related bacteria (85). Moreover, not all susceptible members of the microbiome are equally targeted by SPBs; rather, certain species are more sensitive than others. For example, Subtilosin A, a RiPP produced by *Bacillus subtilis*, had a minimum inhibitory concentration (MIC) of 1.25 μg/ml against *Streptococcus pyogens*, but a MIC of 83.25 µg/ml against *Streptococcus gordonii*, a member of the same genus (86). This property makes SPBs an interesting therapeutic candidate for AD, in which dysbiotic colonization with single bacterial species is present.

SPBs are widely used in food and veterinary medicine. Medical applications in humans have experienced low growth due to insufficient investment. Today, however, there is growing interest in the potential of SPBs (76). A suitable example is *Clostridium difficile*-associated diarrhea (CDAD). It is well known that broad-spectrum antibiotic treatment for *Clostridium difficile* intestinal infections provides acute relief to patients but disrupts the gut microbiome with long-term use by depleting commensal bacteria necessary to control *Clostridium difficile* growth. This altered environment contributes to the thriving of *Clostridium difficile* and the secretion of toxins that cause diarrheal disease (87). In this case, the search for narrow spectrum bacteriocins from the commensal microbiome led to the discovery of a SPB of class RiPP- sactibitoic called thuricin CD, which showed potent inhibition of *Clostridium difficile* without affecting the commensal gut microbiome (88). In addition, the semisynthetic thiopeptide LEF571 has been tested in clinical trials for the treatment of CDAD but showed lower narrow spectrum activity compared with thuricin CD (89). Interestingly, NAI003, a derivative of thiopeptide GE2270A, showed selective activity against *Cutibacterium acnes* over the skin commensals. This thiopeptide has already completed a phase 1 clinical trial for the topical treatment of acne and provides evidence of use of SPBs as topical treatments for skin conditions (90). *Lactocillin*, a thiopeptide SPB isolated from a vaginal commensal *lactobacillus* was shown to inhibit several pathogens colonising skin or vagina and showed no antimicrobial activity against other lactobacilli species (91). Despite increasing research into how SPBs work, the exact reason for their selectivity is still not clear (92). However, this can be attributed to a combination of their properties such as amphipathicity, conformation, charge, hydrophilicity, and secondary structure.

### 4.2 Short peptide bacteriocins in skin microbiome modulation

**Figure 3:**
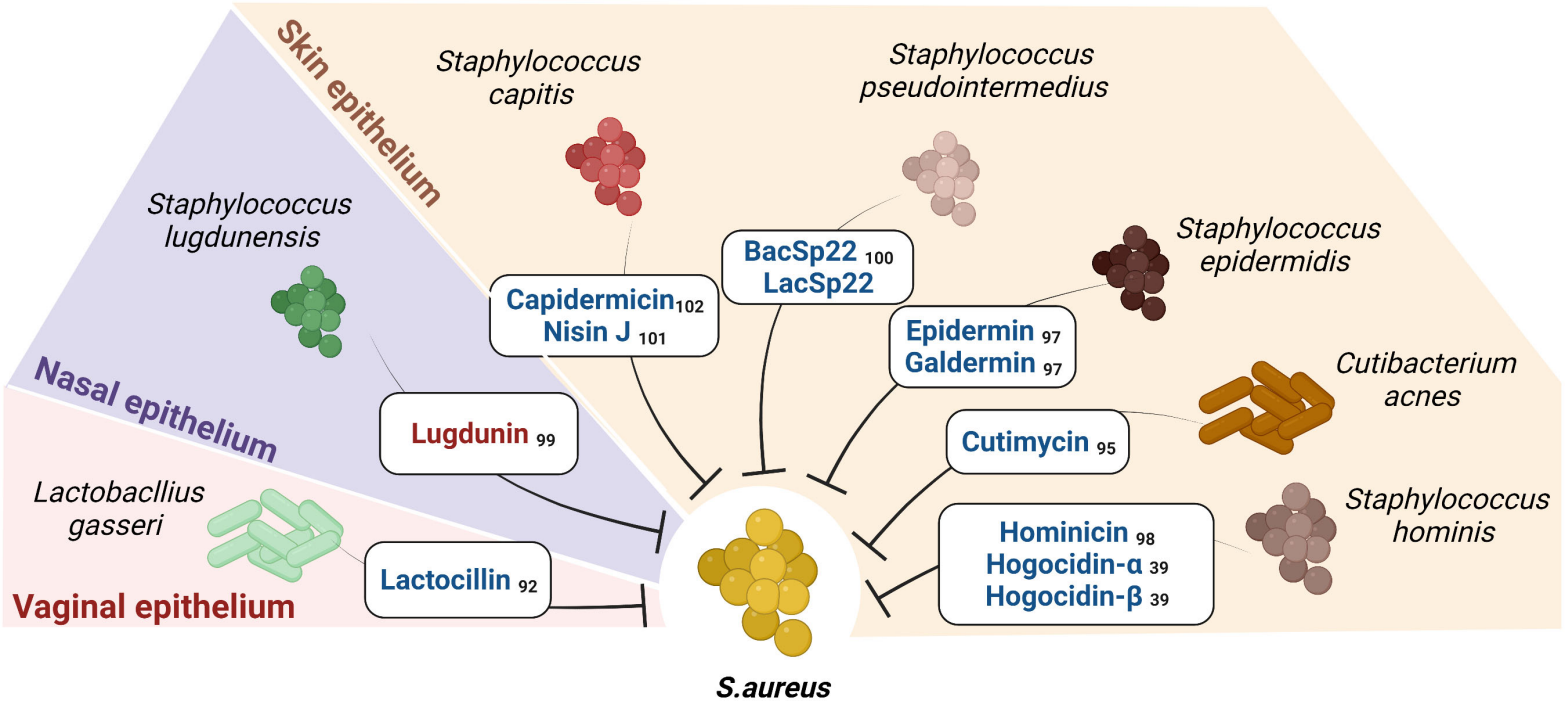
Short Peptide Bacteriocins (SPBs) secreted by certain commensals on human epithelia inhibit *S.aureus*: The figure enlists the SPBs isolated from human epithelial residing commensal bacteria known to possess narrow spectrum activity against *S. aureus* and sparing certain commensals *(*the color red represents NRPs, and color blue represents RiPPs). Subscripts denote references.

Metagenomic analyses revealed that biosynthetic gene clusters encoding bacteriocins are ubiquitous in microbes associated with humans (91). O’Sullivan et al. further demonstrated that the human skin microbiome provides colonization resistance to pathogens by secreting a variety of novel bacteriocins (93). Within the skin microbiome, an inter-genera competition exists (**Figure 3**); for example, *Cutibacterium acnes* secretes a thiopeptide RiPP called cutimycin that inhibits members of *Staphylococcus* but does not affect the members of the genera *Corynebacterium* and *Cutibacterium* (94). However, the mechanism of action remains unknown. Similarly, RiPPs from *lactobacilli* have shown to inhibit members of *Staphylococci*, *Cutibacterium* and *Corynebacterium* (91, 95).

The chances of obtaining bacteriocins with a narrow spectrum of activity are greater if isolated from a phylogenetically similar species or a species that cohabits with the target species. Several staphylococcal-derived bacteriocins exhibit antimicrobial activity against *S. aureus* (96). Known SPBs are epidermin from *Staphylococcus epidermidis* (97), hominicin from *Staphylococcus hominis* (98), lugdunin from *Staphylococcus lugdunensis* (99), BacSp22 from *Staphylococcus Pseudintermedius* (100) and capidermicin *and* nisin J *from Staphylococcus capitis* (101, 102). An approach used by Nakatsuji et al. showed that several CoNS isolated from healthy skin inhibited *S. aureus*. In addition, they isolated and identified the *S. hominis* A9-derived RiPPs *Sh*-lantibiotic-alpha (Hogocidin-α) and *Sh*-lantibiotic-beta (Hogocidin-β), which inhibited *S. aureus* but showed no antimicrobial activity against commensal bacteria namely *Staphylococcus epidermidis* and *Staphylococcus hominis* (39).

### 4.3 Short peptide bacteriocins as immunomodulators

Reports indicate that several SPBs modulate the host immune response (103). In bovine gut epithelium, oral administration of nisin for a short period resulted in an increased accumulation of CD4^+^ and CD8^+^ T lymphocytes (LT) and a decrease in B lymphocytes (104). However, it remains to be determined whether Nisin has as direct effect on epithelial or immune cells or an indirect effect mediated by gut microbiome changes. Interestingly, a higher concentration of nisin was shown to activate extracellular trap release (NETs) and increased intracellular superoxide levels in human neutrophils *in-vitro* (105). In contrast, in another study nisin showed high biological compatibility with explant cultures of rabbit vaginal tissue and did not exhibit immunomodulatory effects (106). This suggests that the immunomodulatory activities of SPBs may be dependent on the epithelia or the tissue under consideration. *Lactobacilli* are endogenous inhabitants of healthy skin. Hemert et al. investigated the immunomodulatory effects of *Lactobacillus Plantarum* (*L. plantarum*) by evaluating its ability to stimulate cytokine production in PBMCs. They found that *L. plantarum* strains stimulated the secretion of the anti-inflammatory cytokines IL -10 and IL -12 more than 10-fold. They moreover identified genetic loci responsible for immunomodulatory capabilities involving components of the bacteriocin biosynthesis and transport pathways (107), suggesting an anti-inflammatory effect of bacteriocins. Interestingly, Thiostrepton an SPB belonging to the thiopeptide RiPP class, was able to inhibit psoriasis-like inflammation induced by TLR7, TLR8, and TLR9 (108). Additionally, Lugdunin, an SPB belonging to the NRP class and produced by the nasal commensal *Staphylococcus lugdunensis*, provided multilevel protection against *S. aureus*. In addition to directly inhibiting *S. aureus*, it also enhanced the innate immune response by recruiting neutrophil granulocytes and monocytes in a mouse model of *S. aureus* infection. Similarly, BacSp222, a RiPP produced by a common skin colonizer, *Staphylococcus pseudintermedius*, showed immunomodulatory and cytotoxic properties apart from its antimicrobial activity (101).

### 4.4 Quorum quenching AMPs against *S. aureus*

**Figure 4:**
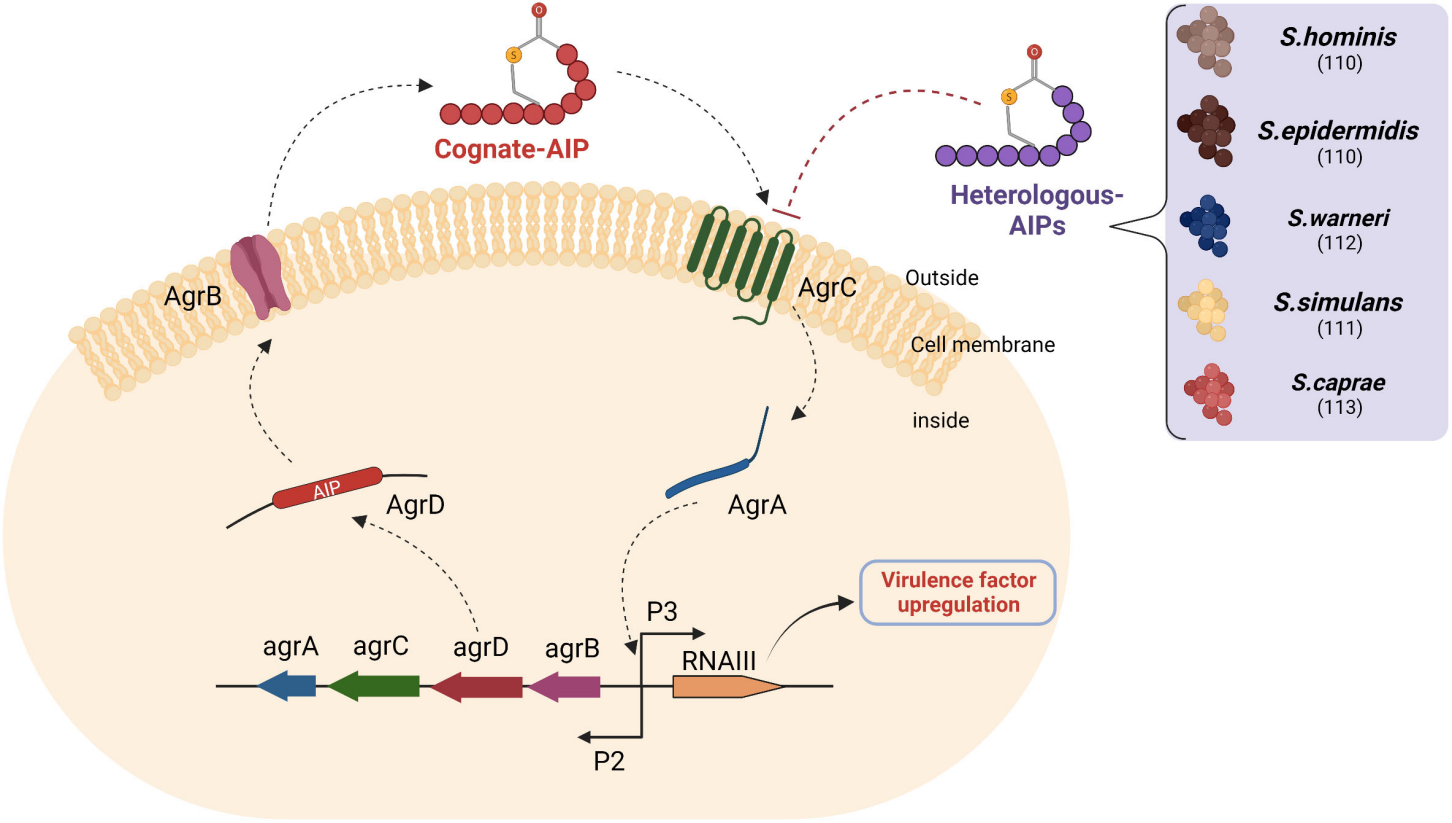
Heterologous AIPs inhibit agr quorum sensing of *S. aureus*: AIPs secreted by *S. aureus* (Cognate AIPs) and recognized *via* the AgrC receptor of the same species activate AgrA-mediated transcription of two divergent transcripts under the control of promoters (P), namely P2 and P3. The P2 promoter encodes the quorum-sensing machinery, while the P3 promoter encodes RNAIII. The RNAIII transcript is responsible for the expression of exoprotein virulence factors. AgrD is a precursor of autoinducing peptide, which is processed by AgrB and then exported to the extracellular space. Several heterologous AIPs secreted by CoNS compete with cognate AIP for binding to AgrC and inhibit downstream Agr signalling. The numbers in brackets denote references. Abbreviations: agr- accessory gene regulation, P2: Promoter region 2, P3: Promoter region 3.

As mentioned earlier, the agr quorum-sensing system of *S. aureus* plays a significant role in the secretion of virulence factors observed in AD (**Figure 4**). The system kicks in when cognate autoinducing peptides bind to a kinase receptor called AgrC. AgrC activates the downstream regulator AgrA, which triggers transcription of the agrBDCA operon and regulatory small RNA called RNAIII by binding to promoter regions P2 and P3. While agrBDCA is responsible for the production of quorum sensing machinery the RNAIII induces transcription of several virulence factors associated to the pathogenesis of AD (35).

The ability of certain bacterial supernatants to modulate the *S. aureus* agr system led to an interest in discovering the metabolites responsible for this phenomenon, which later became known as heterologous AIPs. These heterologous AIPs had similar structures to cognate AIPs (cyclic 7-12 amino acid long with a thiolactone group). This phenomenon is also referred to as quorum quenching and can be used as a therapeutic target in AD (109). Williams et al. identified and isolated an AIP from *S. hominins* with a potent inhibitory effect on the *S. aureus* quorum sensing (110). This AIP successfully inhibited *S. aureus*-mediated epidermal proteolysis and inflammation in mouse skin. Another study showed that synthetic heterologous AIPs identified from *Staphylococcus simulans* isolated from humans and cattle were able to reduce dermonecrotic and epicutaneous skin lesions in mouse models of methicillin-resistant *S. aureus* (MRSA) skin infections by inhibiting all agr quorum sensing signalling subtypes (111). Similarly, several peptide quorum quenchers, including those from *Staphylococcus warneri*, *Staphylococcus capitis*, and *Staphylococcus epidermidis*, have potential as therapeutic agents in AD (110, 112, 113). Interestingly, apicidin, a cyclic fungal tetrapeptide, also inhibited all agr QS systems in methicillin-resistant *S. aureus* (MRSA), suggesting that there is competition within kingdoms that can be exploited in the discovery of new quorum quenching AMPs (114).

The therapeutic potential of quorum quenching molecules is attributed in part to indirect immunomodulatory effects by quorum quenching, i.e., inhibition of Agr-associated virulence factors that interact with immune cells. However, in a recent publication by Pundir et al, many Gram-positive bacterial AIPs were shown to be recognized by the mast cell-specific receptor in humans and mice (Mrgprb2 and MRGPRX2). Among these AIPs, they found that the competence-stimulating peptide secreted by *Streptococcus pneumoniae* (CSP)-1 strongly activated Mrgprb2 and MRGPRX2 and induced effective mast cell degranulation that inhibited bacterial growth and biofilm formation (115).

## 5 Perspectives and challenges

Despite intensive research, a cure for AD is not yet possible. Currently, there are several treatment options for AD but due to the heterogeneous course of the disease, not all patients respond well, therefore there is an urgent need to develop novel treatment strategies. Topical antibiotics have shown little promise as the sole treatment for AD unless secondary infections are involved (116). In addition, treatment guidelines discourage the use of topical antibiotics (117). This is due to the broad-spectrum activity of marketed antibiotics, which not only inhibit *S. aureus* but also kill commensal bacteria, which, as mentioned earlier, are critical for homeostasis and resistance to *S.aureus* colonization. Recently, ATx201 (Niclosamide), a small molecule, was introduced as a promising AD therapy (118). The therapeutic potential was attributed to ATx201’s narrow spectrum activity against *S. aureus* without causing damage to the commensal microbiome (119). Interestingly, inhibition of *S. aureus* growth by live bacteriotherapy in AD has also shown promising results (120, 121). The mediators responsible for this effect were bacterial AMPs of class SPBs with a narrow spectrum of activity. Bacteriotherapy, while promising, also presents some challenges, e.g., we currently lack an understanding of the metabolism and interactions of individual bacteria when exposed to a complex microbial environment, as well as their long-term safety. This is especially true for the changes that result from interactions with mobile genetic elements. Long-term culture of bacteria can lead to spontaneous mutations that result in the loss or gain of undesirable functions. In addition, it is difficult to assess the purity and composition of live bacterial products compared to chemical components.

An alternative strategy is to use well-characterized bacterial metabolites, such as bacterial AMPs, to treat AD. Many SPBs have shown that they can be used in clinical practice (76). To date, however, most of them have been limited to use in animals. Certain patented SPBs, namely lugdunin, Hogocidin-α, and Hogocidin-ß, have already shown narrow-spectrum activity against *S. aureus*, but their therapeutic potential in AD remains uninvestigated. Further research is needed to explore and characterize the vast pool of undiscovered SPBs from the skin microbiome. Inadequate investment and lengthy identification and isolation procedures have severely hindered the development of SPBs as therapeutics. Technological advances in genomics have recently enabled the use of metagenome databases and the identification of novel bacteriocins from biosynthetic gene clusters using genome mining tools (122, 123). Mass spectrometry-assisted peptidomics is another popular technology commonly used to identify novel biomarkers from clinical blood samples for a variety of diseases. Recently, more robust techniques have enabled the application of this approach to a range of biological tissues (124). For example, Azkargorta et al. performed a differential peptidomic analysis of the natural peptide content of the endometrium and demonstrated the presence and activity of antimicrobial peptides *in situ* (125). Whether a similar approach can be used to identify novel bacterial AMPs from skin remains to be investigated but sounds promising.

Several SPBs are active against multidrug-resistant *S. aureus* and are also less likely to develop resistance compared with conventional antibiotics (81). For example, Oyama et al. identified two antimicrobial peptides from the rumen microbiome metagenome data set that are active against multidrug-resistant *S. aureus*. When they examined the likelihood of resistance development after exposure to sub-MIC levels of these peptides, they found that the AMPs did not generate resistant mutants for 20 days. Moreover, the MIC remained within a 1-2-fold increase compared with mupirocin, a marketed topical antibiotic that had a 32-fold MIC increase (126). Nevertheless, there are reports of the development of resistance to the antimicrobial activity of HDPs (127, 128), so the possibility of resistance developing in SPBs cannot be excluded. Therefore, it is important to study the long-term development of resistance of *S. aureus* to SPBs before considering them for therapeutic use. It is known that the addition of SPBs to conventional antibiotics can have a synergistic effect against multidrug-resistant pathogens and also reduces the likelihood of resistance development (129, 130). This raises the likelihood that the use of multiple narrow-spectrum SPBs targeting different mechanisms could have synergistic and favorable microbial killing profiles and reduce the likelihood of resistance development. Some recent studies suggest this phenomenon (131, 132). Alternatively, a combination of bacterial AMPs with HDPs could also show synergistic activities and provide a favorable microbial killing profile, as recently shown by Bitschar et al. (99).

Certain bacterial AMPs have the potential to modulate skin immunity and possess cytotoxic activity (99, 100, 108, 115). On the one hand, this property makes bacterial AMPs of interest to AD, as there is evidence that an inadequate immune response due to a type 2 inflammatory environment and the absence of immune-enhancing cues from the commensal microbiome is a feature of AD (38, 133). On the other hand, it also raises the question of a possible cytotoxic effect of bacterial AMPs on the host. However, it should be kept in mind that bacterial AMPs derived from the human skin microbiome are ubiquitous on the skin and therefore may be better tolerated (91). NAI003, a derivative of a thiopeptide SPB, has completed a phase 1 clinical trial for acne treatment, providing evidence that SPBs can be safely used as topical agents (90). However, it will be extremely important to investigate the chronic effects of such bacterial AMPs at therapeutic concentrations on the skin and their potential to trigger inflammation.

Antimicrobial screening campaigns from the microbiome have so far focused only on the inhibition of pathogens; however, further research should also highlight the ability of bacteriocins to protect commensals. Targeted delivery of antimicrobials and the use of bacteriophages are other promising tools that can be used to kill specific microbes without harming commensals (134, 135).

Another way to render *S. aureus* ineffective is to affect its quorum sensing by bacterial AMPs. Studies targeting quorum sensing of *S. aureus* with synthetic AIPs have shown improved outcomes in AD mouse models and *S. aureus*-associated infections following the reduction of virulence factors. Inhibition of quorum sensing could reduce the production of proteases by *S. aureus* that are responsible for inactivating bacteriocins. This leads us to an interesting question: can a combination of SPBs and a heterologous AIP result in a better therapeutic outcome and a lower risk of resistance development in AD?

It is also important to note that *S. aureus* associated with AD, is non-communicable. This is because the conditions for *S. aureus* colonization and establishment of AD requires (I) altered barrier function (24, 136) or (II) loss of the commensal microbiota necessary to limit *S. aureus* (39), or (III) an inadequate immune response (66, 137) or their combination; all of which are normally intact in healthy individuals.

## 6 Limitations of targeted control of *S. aureus* and its virulence in AD

Several authors have put forward the idea that skin microbiome manipulation with targeted control of *S. aureus* colonization is a therapeutic aim in AD (30, 32, 38), and recent findings strongly support this idea (39, 110, 118, 121). However, it should be noted that not all patients with AD are colonized with *S. aureus*. This suggests that the disease is caused by multiple factors and their interaction with each other. It appears that certain commensal microbes which tend to act as pathobionts can take over the role of *S. aureus* in its absence. For example, *Staphylococcus epidermidis*, a skin commensal, has been shown to colonize the skin of AD and produce proteases that damage the host and induce expression of AD-associated proinflammatory cytokines in human primary keratinocytes (138, 139). However, further research is required to elucidate the fate and role of pathobionts like *Staphylococcus epidermidis* in AD. In addition, host gene defects, e.g., filaggrin, lower HDP expression owing to type 2 cytokine action (66, 140), and environmental factors may play an exacerbating role in AD. Whether therapies targeting *S. aureus* have any effect when *S. aureus* is not involved in AD remains to be determined but seems unrealistic. In such cases, it is important that the microbial AMPs used also target pathogenic *Staphylococcus epidermidis*, possess additional immunomodulatory activities and/or be supplemented with therapeutics that improve the skin barrier or immune system, or both.

In addition, studies using biologics targeting the immune system in AD patients show decreased *S. aureus* abundance after treatment (137, 141), suggesting the bidirectional nature of the disease and supporting the proposition that *S. aureus* is not the initiator of AD but rather a mediator that exacerbates the disease. This also leads to the question if microbial AMPs alone can lead to therapeutic efficacy equal to baseline level, which remains to be investigated.

## 7 Conclusion

Selective killing or virulence inhibition of *S. aureus* without damage to the commensal microbiome is an important therapeutic approach in AD. Evidence suggests that certain bacterial AMPs, which include short peptide bacteriocins and heterologous autoinducing peptides isolated from commensal microbiome, have the potential to treat AD by selectively inhibiting *S. aureus* or its virulence and/or by immunomodulation. This warrants the discovery of novel bacterial AMPs from members of the commensal microbiome of skin surface for the treatment of AD.

## Data availability statement

The original contributions presented in the study are included in the article/supplementary material. Further inquiries can be directed to the corresponding author.

## Author contributions

VP and AJ conceived the ideas. AJ drafted the manuscript and figures. All authors critically revised the manuscript to provide important intellectual content. All authors contributed to the article and approved the submitted version.
